# Visual Characteristics of Adults with Long-Standing History of Dietary Exposure to Mercury in Grassy Narrows First Nation, Canada

**DOI:** 10.3390/ijerph20064827

**Published:** 2023-03-09

**Authors:** Benoit Tousignant, Annie Chatillon, Aline Philibert, Judy Da Silva, Myriam Fillion, Donna Mergler

**Affiliations:** 1 School of Optometry, Université de Montréal, 3744 Jean-Brillant, Montreal, QC H3T 1P1, Canada; 2 Department of Social and Preventive Medicine, School of Public Health, Université de Montréal, 7101 Avenue du Parc, Montreal, QC H3N 1X9, Canada; 3 Centre de Recherche Interdisciplinaire sur le Bien-être, la Santé, la Société et L’environnement (Cinbiose), Université du Québec à Montréal, C.P. 8888, Succ. Centre-Ville, Montréal, QC H3C 3P8, Canada; 4 Grassy Narrows First Nation, General Delivery, Grassy Narrows, ON P0X 1B0, Canada; 5 Département Science et Technologie, Université TÉLUQ, 5800, Rue Saint-Denis, Bureau 1105, Montréal, QC H2S 3L5, Canada

**Keywords:** methyl mercury, visual field, optical coherence tomography, color vision, contrast sensitivity, Indigenous peoples

## Abstract

Since the 1960s, Grassy Narrows First Nation (Ontario, Canada) has been exposed to methyl mercury (Hg) through fish consumption, resulting from industrial pollution of their territorial waters. This cross-sectional study describes the visual characteristics of adults with documented Hg exposure between 1970 and 1997. Oculo-visual examinations of 80 community members included visual acuity, automated visual fields, optical coherence tomography [OCT], color vision and contrast sensitivity. Median age was 57 years (IQR 51–63) and 55% of participants were women. Median visual acuity was 0.1 logMAR (Snellen 6/6.4; IQR 0–0.2). A total of 26% of participants presented a Visual Field Index inferior to 62%, and qualitative losses assessment showed concentric constriction (18%), end-stage concentric loss (18%), and complex defects (24%). On OCT, retinal nerve fiber layer scans showed 74% of participants within normal/green range. For color testing with the Hardy, Rand, and Rittler test, 40% presented at least one type of color defect, and with the Lanthony D-15 test, median color confusion index was 1.59 (IQR 1.33–1.96). Contrast sensitivity showed moderate loss for 83% of participants. These findings demonstrate important loss of visual field, color vision, and contrast sensitivity in older adults in a context of long-term exposure to Hg in Grassy Narrows First Nation.

## 1. Introduction

For many Indigenous communities around the world, fish is an important component of traditional food systems [1]; for some, fish is not only a nutritious food [2], but also the heart of their culture and identity [3,4]. Colonization and dispossession have resulted in land degradation and pollution, with profound impacts on Indigenous food systems [5,6]. Mercury (Hg), from local and global sources, has polluted lakes, rivers, and oceans, bioaccumulating and biomagnifying through the aquatic food chain [7]. While the benefits of fish consumption have been widely documented [8,9], coastal and riverside Indigenous communities who rely on fish for sustenance are often at risk from the toxic effects of methyl Hg exposure [3,10,11]. In Northern Ontario, Canada, the Asubpeeschoseewagong Anishinabek (also known as Grassy Narrows First Nation) people have been exposed to Hg for 60 years through fish consumption. Between 1962 and 1975, a chloralkali plant of a pulp and paper mill discharged almost 10 tons of Hg into the fluvial lake system on their traditional territories. In 1970, fish was a dietary mainstay [12] and blood Hg concentrations were the highest in Canada [13]. Hg exposure decreased over time and stabilized in the mid 1980s [14], paralleling the decline in fish Hg concentrations [15]. Fish Hg levels remain high due primarily to the remobilization of inorganic Hg from riverbank erosion [16].

The visual system is a known target for methyl Hg toxicity from fish consumption. Visual field constriction is a recognized cardinal feature of Minamata disease, a severe neurological disorder first described following high exposure to methyl Hg in the Minamata Bay [17,18,19,20,21,22]. Imaging studies have shown that such visual field constrictions were associated with alterations in the striate cortex [23,24,25], corresponding to the retinotopic mapping of the visual cortex [26]. Studies in communities with lower exposures have revealed methyl Hg-related visual deficits in near visual contrast sensitivity [27,28] and near visual acuity [29], as well as acquired color vision loss [28,30] and early onset age-related cataracts [31]. Methyl Hg-related visual deficits are supported by imaging studies and electrophysiological assessments [23,25,32]. Impaired visual processing through alterations of visual evoked potentials (VEP) [33,34,35,36] has been associated with prenatal Hg exposure. Animal studies provide evidence that methyl Hg acts not only on the central nervous system, but also on the optic nerve and the retina [37,38,39,40]. Monkeys exposed to methyl Hg pre- and postnatally showed later-life reduction of visual functions [41,42].

The objective of the present study is to provide a comprehensive portrait of the eye and visual function characteristics in older adults of Grassy Narrows First Nation, with a history of Hg exposure from freshwater fish consumption.

## 2. Materials and Methods

### 2.1. Study Design

This cross-sectional study is part of an ongoing community–university research partnership to better understand how the long-standing history of methyl Hg exposure has been affecting current health and wellbeing in Grassy Narrows. Participants were selected from the hair Hg biomarker database (1970–1997), previously described [14,43]. Inclusion criteria were having at least four year-based Hg measurements and living in Grassy Narrows community or the immediate region (*n* = 130). Of these, 89 (68.7%) participated in the study. There was no difference in age or sex between participants (median: 57 years; interquartile range: 51.8–63.3) and non-participants (median: 55 years; interquartile range: 51.0–61). Additional inclusion and exclusion criteria were specific to each test and are detailed below.

### 2.2. Oculo–Visual Examination

A total of 81 participants (91% of total) underwent oculo–visual examinations, performed by three therapeutic optometrists, following standard operating procedures. In June and July 2021, assessments were carried out in a community school classroom, converted into an examination room. The examinations included the following assessments:

Visual acuity. Distance visual acuity (DVA) was measured in logMAR using the Early Treatment Diabetic Retinopathy Study (ETDRS) [44] computerized logarithmic letter chart, with participants wearing habitual distance spectacle correction, if any. The measurements were performed monocularly, then binocularly. Pinhole acuity was assessed whenever a monocular measure was 0.3 logMAR or higher (6/12 Snellen equivalent or worse). Near visual acuity (NVA) was measured with the near ETDRS logarithmic chart, first for each eye individually, then with both eyes. Participants wore their habitual spectacle correction used for near activities, when applicable.

Refraction. The HandyRef-K autorefractor (NIDEK CO., Ltd., Gamagori, Japan) was used to measure participants’ refraction. This allowed us to optimize the reliability of other tests, which required adequate near vision, such as color vision, contrast sensitivity, and visual field testing, using appropriate trial lenses.

Optical coherence tomography (OCT). OCT measurements were carried out using the Cirrus HD-OCT device (Carl Zeiss, Meditec Inc., Oberkochen, Germany). Two protocols were performed for each eye, through undilated pupils. The first scan was centered on the optic nerve head (ONH 200 × 200 protocol). The parameters selected for analysis were retrieved from the Cirrus HD-OCT RNFL and ONH Analysis Report and include the average RNFL thickness (aRNFL), the RNFL symmetry percentage, and the RNFL thickness for each quadrant (superior, nasal, inferior, temporal). The second scan was centered on the fovea (macular cube 512 × 128). The parameters selected for analysis were the average ganglion cell layer (GCL) + inner plexiform layer (IPL) thickness, the minimum GCL + IPL thickness, and the average thickness of the GCL + IPL in the six sectors of the elliptical annulus of the thickness map (superior, nasal-superior, nasal-inferior, inferior, temporal–inferior, and temporal–superior), all provided in the Cirrus HD-OCT Ganglion cell Analysis Report. When possible, scan acquisition was repeated in cases with weak signal or acquisition errors (blinking, saccade, misalignment). For analysis, only scans with signal strength of 6/10 or stronger were retained. Scans with persistent acquisition errors, errors in segmentation algorithms, and obvious retinal or optic nerve concomitant pathology visible on the OCT scans were also excluded from analysis.

Cataract grading. Because the presence of significant cataracts has the potential to impact the results of the other variables measured, anterior segment eye examination was performed with a portable slit lamp (PSL Classic portable slit lamp, Keeler Inc., Windsor, UK), through undilated pupils. Cataracts were graded using the WHO cataract grading system [45] for cortical, nuclear, and posterior subcapsular opacities. Considering the results, the clinicians then graded the cataract in terms of visual impact according to clinical judgement (no impact, mild impact, moderate impact, or severe impact) for each eye. Eyes showing cataracts judged to have a severe visual impact were excluded from all analyses.

Color vision. Color vision function was evaluated using three distinct tests: the Hardy, Rand, and Rittler (HRR) Standard Pseudoisochromatic Test, 4th edition (Good-Lite), the Farnsworth D-15 saturated test, and the Lanthony D-15 desaturated test. The tests were administered with True Daylight illuminator (Good-Lite Company, Elgin, IL, USA), with habitual corrective spectacles in place for participants of 39 years and less, and for participants 40 years and more whose near visual acuity was 6/15 or better. Those presenting a near acuity of 6/18 or worse for at least one eye were provided with trial lenses corresponding to the result of the autorefractor, plus 2.00D. The HRR test allows the identification of the type of color defect on all three color axes, and the quantification of the color vision defect as mild, medium, or strong [46]. It has shown to be superior to the Ishihara test for detecting acquired color vision defects in patients presenting optic neuropathies [47]. It has also been suggested as a more practical alternative to the Farnsworth–Munsell 100 Hue test, with comparable ability to detect color vision defects [48]. The test was administered monocularly according to guidelines provided by the manufacturer. As recommended, for the second eye tested, the booklet was rotated upside down from initial position, to reduce the potential memorization. Color vision was also tested binocularly using the Farnsworth saturated D-15 to ensure understanding of the testing procedure. This was followed by the Lanthony D-15 desaturated version, tested monocularly. Both the saturated and desaturated versions of the D-15 required participants to place 15 caps in order of chromatic similarity. The Color Confusion Index (CCI), a ratio based on the sum of chromatic differences between adjacent caps [49], was calculated. A CCI of 1 characterizes a normal color vision; CCI increases as color vision decreases. Results above a threshold of 1.2 are considered abnormal. Plotting tests results on circular D-15 diagrams allows qualitative analysis of the type of color defect (normal, deutan, protan, tritan, tetartan). This test has been widely used for the assessment of acquired color vision deficiency in exposure to neurotoxic substances, including organic Hg [28,30,50]. Participants with results consistent with congenital color vision defects on the saturated D-15 were excluded from the analysis.

Contrast sensitivity. Contrast sensitivity was measured with the Mars numerical contrast sensitivity test, according to instructions provided by manufacturer, under standard lighting (The Mars Perceptix Corporation, Chappaqua, NY, USA). The Mars test uses charts composed of 48 numbers varying in contrast. The contrast of each number, progressing through the chart, decreases by a factor of 0.04 log units [51]. The test was administered with habitual corrective spectacles, if any. Participants over 40 years old with a near acuity of 6/18 or worse were given trial lenses based on autorefraction and age-appropriate addition. For each eye, the test was performed at 40 cm, which is within the 40 cm to 59 cm range recommended for testing medium spatial frequencies, as per the manufacturer’s guidelines. The test was repeated monocularly for an exploratory appreciation of the effects spatial frequency variation: once at 80 cm (low spatial frequencies), then 20 cm (high spatial frequencies).

Visual field assessment. Visual field was assessed using the Humphrey Field Analyzer 3 (HFA, Carl Zeiss, Meditec Inc., Oberkochen, Germany). The HFA Central 30-2 perimetry protocol assesses a grid of 76 points within the central 30° of the visual field. It has been used extensively in the study of neurological conditions, including toxic neuropathies [52]. The Central 30-2 Swedish Interactive Threshold Algorithm (SITA) Fast strategy protocol was administered for each eye. Tests that did not meet the criteria of <15% false positives and <20% fixation losses were repeated. When the criteria were not met after one repetition, the tests were excluded from analysis. To characterize the different configurations of visual field defects, a qualitative assessment of the pattern deviation plots results was performed independently by two examiners (AC and BT); divergences were discussed to reach consensus. The observed patterns were categorized as normal, mild scattered defects, moderate concentric constriction, end-stage concentric constriction, central defects, other localized scotomas, and complex defects (combination of scotomas not qualifying for other categories).

### 2.3. Statistical Analyses

Prior to statistical analyses, all collected data were cleaned and categorized with respect to normative data according to known cut-off values. Because the outcome measurements from a person’s two eyes are usually positively correlated, the appropriate statistical analysis requires accounting for the inter-eye correlation [53]. Within-group inter-eye differences in outcomes were analyzed, using intraclass correlation coefficient (ICC) and Bland–Altman plots to determine whether to use ‘one-eye’ analyses or ‘two-eye’ analyses [54]. ICC values were classified as excellent: values > 0.90; good: >0.75 and ≤0.9; moderate: ≥0.5 and ≤0.75; and poor: <0.5.

Inclusion criteria for statistical analyses included all participants who completed each examination test. Another round of selection was performed for some tests that require specific criteria (see description of tests above). Among the 81 participants, a total of 80 were eligible for analyses.

A series of descriptive statistics (measures of tendency and dispersion, classification of data, and description) were conducted for each measurement, to provide a comprehensive portrait of eye and vision characteristics. Comparisons of score values between sex and with age were analyzed using analysis of variance (ANOVA) (parametric test) or Wilcoxon/Kruskal–Wallis test (rank sums; non-parametric test). When comparing classes (categorical variables), the chi-squared tests (Pearson’s chi-squared test) was used. When comparing contrast sensitivity for various testing distances, the mean difference between two sets of observations was done.

### 2.4. Ethics and Informed Consent

This study was conducted in accordance with the Declaration of Helsinki as well as the OCAP^®^ Principles of Ownership, Control, Access, and Possession developed by the First Nations Information Governance Centre [55]. The study protocol was approved by Grassy Narrows First Nation Chief and Council. Ethics approval was obtained from the Université du Québec à Montréal Research Ethics Board (certificate #3763_e_2020; 9 April 2020) and Manitoulin Anishinaabek Research Review Committee (certificate #2022-06; 26 May 2022). The manuscript was reviewed and approved by Grassy Narrows Chief and Council. Informed consent was obtained from all participants involved in the study.

## 3. Results

The 80 participants included 44 (55%) women and 36 (45%) men. Median age was 57 years (IQR 51.3–63.8). ICC and Bland–Altman plots showed a good level of inter-eye agreement (most variables < 0.75). Therefore, based on random selection, a total of 40 right eyes (23 female, 17 male) and 40 left eyes (21 female, 19 male) were used for statistical analyses.

### 3.1. Visual Acuity

Table 1 shows the characteristics of DVA and NVA. When including optimized pinhole VA (*n* = 32) to account for uncorrected refractive error, median DVA was of 0.1 (Snellen 6/6.4; IQR 0–0.2). Binocular presenting NVA was 0.3 logMAR (6/12 Snellen; IQR 0.3–0.5). There was no influence of age or sex on VA.

### 3.2. Automated Visual Field

Distribution of global indices for visual field are reported in Table 2. At least half of participants presented abnormal values (P < 0.5%) for mean deviation (MD) and/or pattern standard deviation (PSD) indices, consistent with the presence of overall field depression or localized scotomas, respectively. One-fourth (25.7%) of participants presented a defect using the 62% of VFI as a cut-off value for moderate visual field loss [56].

No differences were observed with respect to sex for global indices in visual field results. However, more older participants presented abnormal values for MD and more were outside normal limits for GHT than the younger ones (Wilcoxon/Kruskal–Wallis Tests [Rank Sums] 1-Way Test, chi-square Approximation, χ^2^ = 8.11, *p* = 0.030 and χ^2^ = 13.5, *p* = 0.017, respectively).

**Figure 1:**
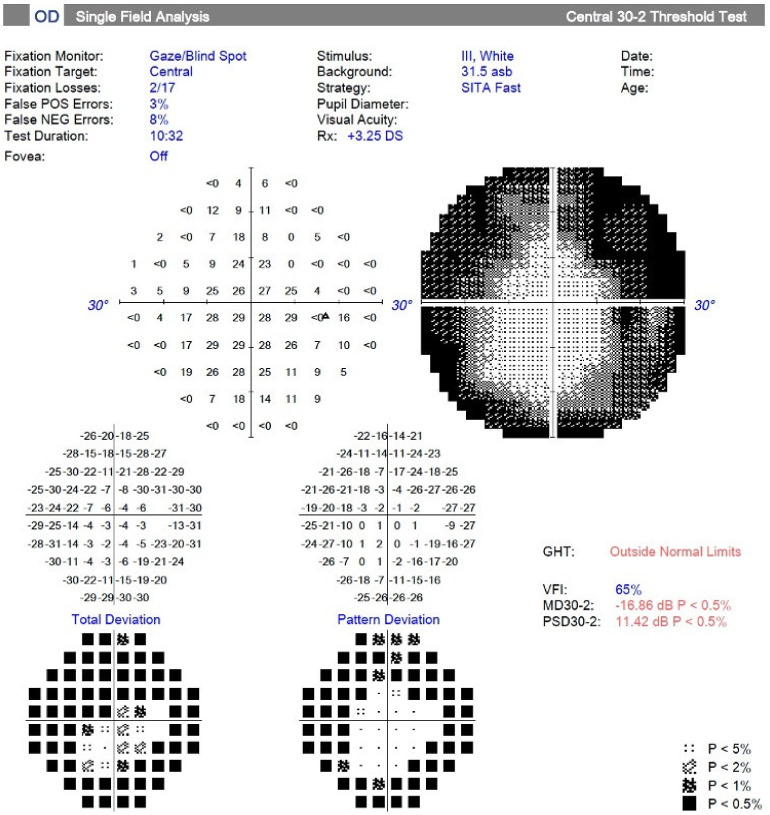
Printout of automated visual field testing showing concentric constriction.

Qualitative assessment of pattern deviation plots showed that three-quarters of participants had a visual field with defects, the most common being concentric and complex defects. An example of concentric peripheral loss of light sensitivity is shown in Figure 1 in the gray-scale plot, along with both mean deviation and pattern deviation plots.

The median number of scotomas in total deviation and in pattern deviation plots were 13 (*n* = 69, IQR: 1–61) and 5 (*n* = 54, IQR: 0–18), respectively. A total of 16 pattern deviation plots (21.7%) were missing because of complete scotomas.

### 3.3. Optical Coherence Tomography

Thickness of retinal layers, as measured by optic nerve and macular OCT scans, are presented in Table 3. The average thicknesses of retinal nerve fiber layer (aRNFL) and macula’s “average GCL + IPL” (ganglion cell layer added to inner plexiform layer) were outside the normal ranges (green color code) for one-fourth of the participants (25.7% and 25.0%, respectively). The minimum GCL + IPL thickness was outside the normal range for almost one-third of participants (29.4%).

No association was found between thicknesses in retinal or macular scans and sex. However, in quadrants analyses, the superior, inferior, and average retinal layers thicknesses decreased with age. Older participants presented more abnormal ranges for superior and inferior retinal layers (Wilcoxon/Kruskal–Wallis Tests (Rank Sums) 1-Way Test, chi-square, χ^2^ = 6.77, *p* = 0.033 and χ^2^ = 8.85, *p* = 0.031, respectively) than younger ones. All thicknesses in macula decreased with age, except for temporal quadrants. Upon qualitative appreciation of the scans, there was no specific or localized pattern of thinning of retinal layers (i.e., superior–inferior asymmetry, nerve fiber bundle loss) consistent with known ocular pathologies.

### 3.4. Color Vision

Table 4 shows that half of the participants had no defect on HRR color vision testing. Blue–yellow and red–green color defects were observed for more than one-third of participants (37.1% and 37.7%, respectively). More women presented a blue–yellow defect than did men (contingency table, χ^2^ = 4.56, *p* = 0.033). One-third of participants had a defect on both color ranges (32.5%). With increasing age, participants presented more defects (Wilcoxon/Kruskal–Wallis Tests (Rank Sums) 1-Way Test, χ^2^ = 10.9, *p* = 0.012).

Monocular desaturated D-15 color vision tests showed that 87% of participants presented a CCI value equal or greater than the abnormal threshold of 1.2. This proportion was 55% for binocular saturated testing. CCI increased with age, for both saturated and desaturated D-15 testing. Qualitative analysis of D-15 graphic plots (not shown here) showed that most participants (*n* = 64, 88%) had complex color vision defects (three or more error lines non-parallel to protan, deutan, tritan or tetartan lines). Sex was not significantly associated with results on saturated and desaturated CCI.

### 3.5. Cataracts

Slit lamp assessment of crystalline lenses showed low degrees (grade 0 or 1) of nuclear sclerosis, cortical opacities, or posterior subcapsular cataracts. More than 89% of combined lens opacities were judged as having either no or mild visual impact.

### 3.6. Contrast Sensitivity

Table 5 shows that contrast sensitivity (CS) thresholds using the Mars chart was abnormal for the great majority of participants (more than 80%) for all spatial frequencies. CS was significantly higher in low (20 cm) and medium spatial frequencies (40 cm) when compared with high (80 cm) spatial frequencies (paired sample *t*-test, t = 3.69, *p* < 0.001; t = 3.12, *p* = 0.003, respectively). There was no significant difference in CS between high and medium spatial frequencies. With increasing age, more participants had severe loss of CS for 80 cm (low spatial frequencies) (Wilcoxon/Kruskal–Wallis Tests [Rank Sums] 1-Way Test, χ^2^ = 10.7, *p* = 0.005).

## 4. Discussion

This study is the first comprehensive description of eye and visual characteristics in a First Nation population of older adults with a documented history of long-standing methyl Hg exposure from fish consumption. Results show that there are abnormalities in visual function of various degrees.

Automated visual field testing revealed that although two-thirds of participants had a VFI of more than 81%, only one-fourth of participants presented normal pattern deviation plots. More than half of participants showed either complex defects or moderate or end-stage concentric visual field constrictions. Although many scotomas were more frequent in participants 50 years and older, half of the younger participants (37–50 years) presented moderate to severe visual field loss. These findings are consistent with previous studies, where concentric constriction in visual field has been widely reported after methyl Hg exposure from dietary intake [24,25,57,58,59,60,61,62,63,64].

In previous studies carried out in Grassy Narrows First Nation, visual field defects were documented using Forster’s perimeter. Harada et al. reported 21.9% of 73 persons examined had a “narrowed visual field” [61]. Takaoka et al. measured visual fields by confrontation visual field test and reported 14% of 44 participants presented with “visual constriction” [65]. In the present study, documentation of visual field using threshold testing with an automated perimeter provided the means to better qualify and quantify these visual losses.

Some of the underlying mechanisms of peripheral visual field defects in a context of long-term methyl Hg toxicity have been described among patients with Minamata disease. This condition, first described in Japan in the 1950s, following ingestion of marine products contaminated with methyl Hg, is characterized by multiple neurological and visual impairments, including visual field loss [59]. Following the classic retinotopic mapping of the visual cortex [26], the central part of the visual field is represented in the posterior part of the striate cortex, while the periphery corresponds to its anterior part, along the calcarine fissure. Among Minamata patients, microscopic examination showed neuropathological lesions including disintegration and loss of neurons in the striate cortex [66]. Magnetic resonance imaging (MRI) scans showed dilatation of the calcarine sulcus caused by atrophy of the visual cortex [23,25,67], and was correlated with visual field losses [25].

The findings of the present study do not resemble the clinical picture of more common conditions causing constricted visual field, such as glaucoma or retinitis pigmentosa. Those diseases are not known to present in such prevalence and clustering pattern in any given population. Glaucoma visual field loss is typically accompanied by specific retinal nerve fiber loss along anatomical nerve fiber bundles corresponding to neural retinal rim loss [68], none of which are obvious in these participants. Retinitis pigmentosa typically presents with pronounced symptoms of nyctalopia and present in familial patterns due to genetic transmission (autosomal dominant, autosomal recessive or X-linked) [69].

Although the current study took place 10 years after the last study looking at visual field of individuals in Grassy Narrows [61], it is noteworthy that, in both studies, visual field constriction was highly prevalent in individuals between 40 and 60 years old. While this does not represent a formal longitudinal assessment, this could, nonetheless, suggest that the effects of methyl Hg develop or endure over a long time period. While biomarkers of Hg exposure have decreased over time in Grassy Narrows [14], Hg-induced impairments of visual function might not recover following a reduction of exposure [70].

This study is one of the first to include retinal thickness data from OCT scans in the context of methyl Hg exposure. Our results show that superior, lower, and average RNFL thicknesses and most macular thicknesses (ganglion cell complex and inner plexiform layer) are lower than the normal range. Quadrant and sectoral thicknesses show no conspicuous areas of predilection for abnormal values, suggesting non-specific thinning. Animal studies have shown retinal accumulation of inorganic Hg [40,71,72] and human studies have reported slight generalized thinning of retinal layers following exposure to inorganic Hg [73,74]. Our results could indicate that visual disturbances following exposure to methyl Hg may be associated, at least in part, to a retinal mechanism. However, the pathophysiology is likely multifactorial and distinct from other disease processes affecting retinal structure and visual field loss, such as glaucoma, where a large loss proportion of retinal fibers, visible on OCT, typically precedes visual field involvement [75].

An alternative hypothesis to the thinner retinal layers could be the involvement of genetic factors. The distribution and thickness of retinal layers has been shown to differ with respect to ethnicity [76,77]. The normative database included in the Cirrus HD-OCT does not include the ethnic characteristics of the Anishinaabe or other First Nation peoples. However, our results are unlikely affected only by potential ethnic variations in retinal layer thickness. When compared to the seven population normative databases in those studies, our sample’s aRNFL measurements were thinner than that of six other ethnicities (signed-rank Wilcoxon tests, *p* < 0.0001) except for that of a population study conducted in India (*p* = 0.32), which had the lowest thickness values of all [76,78].

Color vision testing with desaturated D-15 showed a high proportion of abnormal results (87% of participants above or greater the normal CCI value of 1.2), no sex differences, and a high proportion (88%) of complex patterns of defects. This is inconsistent with congenital color vision loss [79] and points towards acquired color deficiency. This has been reported with exposure to methyl Hg from fish consumption [28], which showed mean CCI values ranging from 1.46 to 1.50 [30,80]. HRR color vision testing also revealed a high proportion (39%) of color vision defects, most of them complex (combined red–green and blue–yellow defects). Although this proportion is inferior to desaturated D-15 defects, it is noteworthy that the HRR testing uses a shorter, forced-choice method compared to the longer, more nuanced ranking task compared to the D-15. This may lead to more specific and less sensitive results with HRR than with D-15, which may be more subject to fatigue and lead to more frequent abnormal results. A number of studies have also shown that persons exposed to methyl Hg may do well on tests using saturated colors such as the Ishihara plates, but performance on desaturated tests is dose-related [19,30,81], and persistent even following reduction of exposure [29].

In this study, contrast sensitivity (CS) was often reduced, with most participants showing moderate to severe contrast loss in medium, low, and high spatial frequencies. These findings are similar to those of previous studies carried out on methyl Hg toxicity using sinusoidal grating tests [27,28,82,83]. Methyl Hg-related decrease in CS was first observed with visual evoked potential testing in patients suffering from Minamata disease in Japan [27]. Some authors suggested alteration of CS function could be connected to methyl Hg accumulation in the retina [39,71,72], or as a result of alterations in the visual cortex, where processing of contrast occurs [19,84,85,86]. Developmental exposure and adulthood exposure to methyl Hg may produce different the patterns of spatial and temporal vision defects [42].

Distance visual acuity (DVA) was in the normal range for most participants in this study. The increase in acuity after pinhole suggested most loss on presenting DVA would be due to uncorrected distance refractive error. These results were expected, as low DVA is not a typical clinical feature of Hg exposure [29,81]. While near visual acuity (NVA) presenting VA were low (0.3 logMAR binocular, 0.5 logMAR monocular), no refractive nor pinhole compensations were performed at near, making it challenging to isolate the influence of presbyopia from these results. One study [29] showed an association between acceleration of the progression of presbyopia in individuals forty years and over and hair Hg concentration.

Considering the nature of this community-based study, we had to make choices about the type and quantity of assessments. Some examinations, such as VEPs or fundoscopy, which could ideally complete a clinical picture in a typical health care setting, were not collected. Rather, emphasis was put on the most known visual characteristics in relation to dietary Hg exposure, notably visual field constriction. The introduction of OCT to an observational study in this context was useful to describe retinal involvement, while helping to distinguish visual losses, such as constricted visual field defects, from other posterior segment conditions such as glaucoma.

## 5. Conclusions

Our findings clearly show that, in this First Nation community, there is a high prevalence of visual anomalies, such as peripheral visual field constriction, reduced retinal thickness, defects in color vision, and reduced contrast sensitivity. Future studies will examine the contribution of long-term Hg exposure from fish consumption and systemic conditions to the visual deficits observed in adults of Grassy Narrows First Nation.

## Figures and Tables

**Table 1:** Characteristics of visual acuity measurements.

	N	Median logMAR (IQR)
Distance visual acuity (presenting)		
Crude monocular (without pinhole)	78	0.2 (0.1–0.27)
Optimized monocular (adjusted with pinhole for *n* = 32)	80	0.1 (0–0.2)
Binocular	80	0.1 (0–0.2)
Near visual acuity (presenting)		
Binocular	80	0.3 (0.3–0.5)

Abbreviations: IQR, interquartile range; LogMAR, logarithm of minimum angle of resolution.

**Table 2:** Distribution of automated visual field indices and qualitative analyses of scotomas.

Visual Field Indices/Analyses	N	Median (IQR)/*n* (%)
Mean Deviation (MD)	68	−5.59 (−17.3; −2.36)
MD < −12 dB	68	21 (31%)
MD P < 0.5%	68	34 (50%)
Pattern Standard Deviation (PSD)	70	5 (2.27–7.81)
PSD P < 0.5%	70	37 (53.6%)
Visual Field Index VFI (%)	70	92 (63–98)
VFI > 81%		48 (64%)
VFI < 62%		18 (25.7%)
VFI < 15%		6 (8.57%)
Glaucoma Hemifield Test (GHT)	75	
Within normal limits		17 (24%)
Borderline		5 (7%)
Outside normal limits		48 (69%)
Qualitative assessment (based on pattern deviation plot)	68	
Normal		16 (23.5%)
Mild scattered defects		8 (17.8%)
Moderate concentric constriction		12 (17.6%)
End-stage concentric constriction		12 (17.6%)
Complex defects		15 (23.5%)
Central defects		1 (1.47%)
Other localized scotomas		3 (4.41%)
Scotomas (more than three contiguous points with P < 0.5%)		
Total deviation plot	68	45 (66%)
Pattern deviation plot	54	27 (51%)

Abbreviations IQR: interquartile range.

**Table 3:** Distribution of retinal layer thicknesses measured by optical coherence tomography (OCT).

OCT Scans	N	Median Thickness μm (IQR)	Measurement in Green/Normal Range *n* (%)
Optic Disc Cube			
Average RNFL thickness	70	86.5 (79–92)	52 (74.3%)
Thickness (by quadrant)			
Superior	70	103 (94–114)	57 (81.4%)
Nasal	70	70.5 (62.8–77)	62 (88.5%)
Inferior	70	102 (103–125)	56 (80%)
Temporal	70	54 (46–61)	56 (80%)
Macular Cube			
Average GCL + IPL thickness	68	78 (72–82)	51 (75%)
Minimum GCL + IPL thickness	68	74.5 (66.3–79)	48 (70.6%)
Thickness (by section)			
Superior	68	78 (72.3–83.8)	52 (76.5%)
Superior nasal	68	79 (74.3–85.8)	56 (82.3%)
Inferior nasal	68	77 (72–83)	54 (79.4%)
Inferior	68	76 (70–81)	54 (79.4%)
Inferior temporal	68	78 (72–83)	55 (80.8%)
Superior temporal	68	77 (72–83)	54 (79.4%)

Abbreviations: RNFL, retinal nerve fiber layer; GCL, ganglion cell layer; IPL, inner plexiform layers.

**Table 4:** Distribution of color vision testing results.

Color Vision Test	N	*n* (%)/Median (IQR)
**HRR testing (monocular)**		
B–Y defect	77	27 (37.1%)
R–G defect	77	29 (37.7%)
Color defect categories	77	
B–Y normal and R–G normal		46 (49.7%)
B–Y normal and R–G defect		4 (5.20%)
B–Y defect and R–G normal		2 (2.60%)
B–Y defect and R–G defect		25 (32.5%)
**D-15 testing**		
Saturated, CCI (binocular)	80	1.17 (1–1.46)
CCI > 1.2		44 (55%)
Desaturated, CCI (monocular)	77	1.59 (1.32–1.96)
CCI > 1.2		67 (87%)

Abbreviations: HRR, Hardy Rand and Rittler test; B–Y, blue–yellow; R–G, red–green; CCI, Color confusion index.

**Table 5:** Distribution of Mars contrast sensitivity at various testing distances.

	N	Median (IQR)/*n* (%)
20 cm (high spatial frequencies)	76	
log CS		1.48 (1.36–1.64)
Categories		
Normal		8 (10.5%)
Moderate		65 (85.5%)
Severe		3 (3.95%)
40 cm (medium spatial frequencies)	76	
log CS		1.48 (1.32–1.67)
Categories		
Normal		10 (13.2%)
Moderate		63 (82.9%)
Severe		3 (3.9%)
80 cm (low spatial frequencies)	76	
log CS		1.44 (1.29–1.56)
Categories		
Normal		7 (9.2%)
Moderate		63 (82.9%)
Severe		6 (7.9%)

Abbreviations: CS, contrast sensitivity.

## Data Availability

Restrictions apply to the availability of these data. Data were obtained from and are the property of the Grassy Narrows First Nation, in keeping with the First Nations principles of Ownership, Control, Access, and Possession (OCAP).
